# Curcumin effects on Leydig cell functions and potential therapeutic uses

**DOI:** 10.1530/EO-22-0075

**Published:** 2023-02-02

**Authors:** Trinidad Raices, María Luisa Varela, Adriana María Belén Abiuso, Elba N Pereyra, Carolina Mondillo, Omar P Pignataro, María Fernanda Riera

**Affiliations:** 1 Laboratory of Molecular Endocrinology and Signal Transduction, Instituto de Biología y Medicina Experimental (IBYME-CONICET), Buenos Aires, Argentina; 2 Centro de Investigaciones Endocrinológicas ’Dr. César Bergadá’ (CEDIE-CONICET-FEI), División de Endocrinología, Hospital de Niños Ricardo Gutiérrez, Buenos Aires, Argentina; 3 Departments of Neurosurgery, and Cell and Developmental Biology, University of Michigan, School of Medicine, Ann Arbor, Michigan, USA

**Keywords:** Leydig cells, curcumin, steroidogenesis, natural therapy

## Abstract

Curcumin has been ascribed with countless therapeutic effects, but its impact on testicular function has been scarcely researched. Leydig cells comprise the androgen-secreting population of the testis and may give rise to Leydig cell tumours (LCTs). Due to their steroid-secreting nature, LCTs entail endocrine, reproductive, and psychological disorders. Approximately 10% are malignant and do not respond to chemotherapy and radiotherapy. The aim of this study was to assess curcumin’s impact on Leydig cells’ functions and its potential effect on LCT growth. *In vitro* assays on MA-10 Leydig cells showed that curcumin (20–80 µmol/L) stimulates acute steroidogenesis, both in the presence and absence of db-cAMP. This effect is accompanied by an increase in StAR expression. Regarding curcumin’s *in vitro* cytostatic capacity, we show that 40–80 µmol/L curcumin reduces MA-10 Leydig cells’ proliferative capacity, which could be explained by the arrest in G2/M and the reduced viability due to the activation of the apoptotic pathway. Finally, CB6F1 mice were inoculated with MA-10 cells to generate ectopic LCT in both flanks. They received i.p. injections of 20 mg/kg curcumin or vehicle every other day for 15 days. We unveiled curcumin’s capacity to inhibit LCT growth as evidenced by reduced tumour volume, weight, and area under the growth curves. No detrimental effects on general health parameters or testicular integrity were observed. These results provide novel evidence of curcumin’s effects on the endocrine cell population of the testis and propose this natural compound as a therapeutic agent for LCT.

## Introduction

*Curcuma longa* L. or turmeric is a plant chiefly cultivated in Southeast Asia with a leading role in folk medicine. Turmeric is also the name of the extract obtained from the rhizome of this plant, which is used as a culinary spice in preparations such as curries and mustard sauce and as a dietary supplement due to its beneficial health effects (Hewlings & Kalman 2017). This extract contains 2–9% of curcuminoids: a group of compounds such as curcumin, demethoxycurcumin, bis-demethoxycurcumin, and cyclic curcumin. Among these, curcumin (1,7-bis(4-hydroxy-3-methoxyphenyl)-1,6-heptadiene-3,5-dione) is the main component. Since the 1990s, when the first pieces of evidence on curcumin’s anticancer potential were reported, there has been a steep increase in curcumin-related publications (Priyadarsini 2014). A myriad of therapeutic effects on a wide range of ailments has been reported ever since. The mechanisms through which this phytochemical exerts its effects are equally diverse, being its antioxidant and anti-inflammatory potential the most relevant (Patel *et al.* 2020).

Leydig cells comprise the interstitial androgen-secreting population of the testis, which displays a steroidogenic response in the presence of pituitary gonadotropins. One of the rate-limiting steps of steroid hormone synthesis involves the transport of cholesterol from the outer to the inner mitochondrial membrane by the steroidogenic acute regulatory (StAR) protein. The main steroid secreted as a result of this process is testosterone, which plays a central role in external genitalia differentiation, reproductive functions, and secondary sex characteristics development (Stocco 2001).

Dysregulation of the population of Leydig cells can result in the development of Leydig cell stromal tumours, which represent about 1–3% of testicular neoplasms in adults and 3% in children. They have two peaks of incidence: in the third to sixth decade in adults, and in children aged 3–9 years (Albers *et al.* 2017). Leydig cell tumours are also an unusual form of ovarian neoplasms (Sachdeva *et al.* 2008). Due to the steroid-secreting nature of the tumours, symptoms include precocious puberty, hormonal and reproductive disorders, and gynecomastia (Pozza *et al.* 2019). Approximately 10% of Leydig cell tumours are malignant and do not respond to chemotherapy and radiotherapy. In these cases, the treatment consists of orchidectomy and early retroperitoneal lymphadenectomy to prevent metastases and achieve a long-term cure. The testis has shown to be highly susceptible to the toxic effects of radio- and chemotherapy at all stages of life, being the prepubertal testes the most vulnerable (Al-Agha & Axiotis 2007). No effective treatment is currently available for metastatic Leydig cell tumours and most treatment protocols are based on the limited evidence provided by case reports. In view of this scenario, it is evident that there is an imperative need for new therapeutic alternatives to be researched.

Considering the scarce evidence of the direct effects of curcumin on the steroidogenic population of the testis, and the vast number of reports proving curcumin’s anticancer potential, the aim of the present report is to assess curcumin’s impact on Leydig cells functions *in vitro* and its potential *in vivo* effect on Leydig cell tumour growth.

## Materials and methods

### Materials

Curcumin was purchased from Cayman Chemical (item No. 81025). Cell culture supplies were obtained from Gibco (Thermo Fisher Scientific) and plasticware from BD Biosciences. [^3^H]-thymidine (20 Ci/mmol) and [^1,2,6,7-3^H]-progesterone were purchased from New England Nuclear Corporation (North Billerica, MA, USA). StAR antibody (item No. FL-285) was from Santa Cruz Biotechnology Inc., β-tubulin antibody (item No. 05-661-I) was from Sigma-Aldrich, and peroxidase-conjugated anti-mouse (item No. PI-2000-1) and anti-rabbit (item No. PI-1000-1) IgG antibodies from Vector Labs (Burlingame, CA, USA). WST-1, Trypan blue, propidium iodide, sodium citrate, RNase A, activated charcoal, dextran, and progesterone antibody were purchased from Sigma-Aldrich.

## Methods

### Culture of MA-10 Leydig tumour cells

MA-10 cell line (ATCC CRL-3050) is a murine clonal strain of Leydig tumour cells, kindly provided by Mario Ascoli (University of Iowa, Iowa City, IA, USA). MA-10 cells were grown in T75 flasks in DMEM/F12 containing 4.76 g/L of HEPES, 1.2 g/L of sodium bicarbonate, 1 mL/L of Gentamicin Reagent Solution, and 15% v/v horse serum at 37°C and 5% CO_2_. After detachment by trypsinization, desired cell concentration was calculated, and they were plated in microplates. Cells were grown for 48 h in complete medium and then treated in serum-free medium. Treatment schemes are specified in the Results section and in each figure legend. Assays were performed with at least three different cell line batches or passage number (<20) of cells and similar results were obtained.

### Real-time PCR (RT-qPCR)

Total RNA was isolated from MA-10 Leydig cell pellets with TRI Reagent (SigmaAldrich) according to the manufacturer's recommendations. The amount of RNA was estimated by spectrophotometry at 260 nm. Reverse transcription (RT) was performed as previously described (Rindone *et al*. 2018). Real-time PCR was performed using Step One Real Time PCR System (Applied Biosystems). Table 1 shows the sequence of the primers used. Amplification was carried out as recommended by the manufacturer: 25 µL reaction mixtures contained 12.5 µL of SYBR Green PCR Master mix (Applied Biosystems), the appropriate primer concentration and 1 µL of cDNA. The relative cDNA concentrations were established by a standard curve using sequential dilutions of a cDNA sample. The amplification program included the initial denaturation step at 95°C for 10 min, 40 cycles of denaturation at 95°C for 15 s and annealing and extension at 60°C for 1 min. Fluorescence was measured at the end of each extension step. After amplification, melting curves were acquired and used to determine the specificity of PCR products. Relative gene expression was calculated using the relative standard curve method. The data were normalized to GAPDH.

**Table 1:** Mouse-specific primers for RT-qPCR.

Gene	Primer sequence (5’→ 3’)		Product size (bp)
	Forward	Reverse	
GAPDH (*Gapdh*)	GGGTGTGAACCACGAGAAAT	ACTGTGGTCATGAGCCCTTC	135
STAR (*Star*)	CCGGAGCAGCGTGGTGTCA	CAGTGGATGAAGCACCATGC	63
p21^Cip1^ (*Cdkn1a*)	GTCTTGCACTCTGGTGTCTCA	GCACTTCAGGGCTTTCTCTT	147
Cyclin D1 (*Ccnd1*)	CTACCGCACAACGCACTTTC	AAGGGCTTCAATCTGTTCCTG	151
p27^Kip1^ (*Cdkn1b*)	TTCGACGCCAGACGTAAAC	TTCAATGGAGTCAGCGATATG	126

### Progesterone radioimmunoassay

Progesterone was measured by radioimmunoassay (RIA) as previously described (Raices *et al.* 2019). Cells were plated in 12-well microplates (1 × 10^5^ cells/well). After the treatments, supernatants were collected for progesterone quantitation. Briefly, 100 μL of the supernatants or standards were incubated overnight with a progesterone antibody (1:5000) and [^3^H]-progesterone. A 10-min incubation with activated charcoal 0.5% w/v and dextran 0.05% w/v was followed by a 15-min centrifugation at 3000 ***g***. Radioactivity was measured in the supernatants using a PerkinElmer Tri-carb 2800TR liquid scintillation analyser.

### Western blot and immunodetection of proteins

Cells were cultivated and treated under the same conditions as those for RIA. Western blot analysis and immunodetection of proteins were carried out as previously described (Piotrkowski *et al.* 2009). StAR antibody dilution was 1:100. β-tubulin antibody (1:5000) was used for equal loading correction. A biotinylated secondary anti-rabbit antibody and the Vectastain ABC Kit (Vector Labs) were used for detection. The optic density of immune-specific bands was quantified using the ImageJ Software (NIH).

### Reactive oxygen species determination

Cellular reactive oxygen species (ROS) were measured by monitoring the oxidation of dichloro-dihydro-fluorescein diacetate (DCDHF-DA) to the fluorescent compound 2’, 7’-dichlorofluorescein (DCF). After the treatments, cells grown in 96-well microplates (5 × 10^3^ cells/well) were washed with PBS and incubated with 25 µmol/L DCDHF-DA in serum-free medium in the dark (30 min, 37°C). Then, cells were washed with PBS and lysed in a hypotonic lysis buffer (KCl 7.5 mM, 1 h, 37°C). Cleared lysates were obtained by centrifugation (4000 ***g***, 20 min, 4°C). Fluorescence intensity was measured in a FLUOstar OPTIMA fluorometer (BMG LABTECH, Ortenberg, Germany) (k_ex_: 485/12 nm; k_em_: 520 nm). Values were normalized to protein concentration determined by Bradford assay (Bradford 1976).

### [^3^H]-Thymidine incorporation assay

Cell proliferation was assessed as a function of [^3^H]-thymidine incorporation into DNA. MA-10 cells were plated in 48-well microplates (2.5 × 10^4^ cells/well) and treated for 24 h. During the last 16 h of treatment, 0.25 μCi/well [^3^H]-thymidine were added. Cells were washed twice with ice-cold PBS. Subsequently, cells were released by trypsinization and harvested by vacuum aspiration onto glass-fibre filters, which were washed, dried, and transferred to glass vials containing a scintillation cocktail (Optiphase Hisafe III scintillation liquid; Wallac, Gaithersburg, MD, USA) for the measurement of radioactivity (Tri-carb 1600TR; Packard, Meriden, CT, USA).

### Cell cycle analysis by flow cytometry

Cell cycle distribution was analysed by DNA content quantitation. Cells were plated in 12-well microplates (1 × 10^5^ cells/well). Following treatments, they were harvested by trypsinization, washed and fixed with 75% v/v ethanol overnight at −20°C. The following day, cells were washed and incubated with 40 μg/mL propidium iodide, 3.8 mmol/L sodium citrate, and 100 μg/mL RNAse A for 30 min at RT. The intracellular propidium iodide fluorescence intensity was measured using a flow cytometer FACSCanto II (BD Biosciences, Franklin Lanes, NJ, USA) (10000 events/sample) and analysed with FlowJo software (BD Biosciences).

### WST-1 assay

Cell proliferation was also assessed indirectly using WST-1 reagent following the manufacturer’s guidelines. Cells were plated in 96-well microplates (5 × 10^3^ cells/well). After the treatments, WST-1 was added to each well (10 µL/well) and a 4-h incubation was performed at 37°C and 5% CO_2_. After mixing, absorbance at 420 nm was measured using a Multiskan FC microplate reader (ThermoScientific).

### Trypan blue exclusion assay

Cell viability was assessed using a vital staining technique. Cells were plated in 12-well microplates (1 × 10^5^ cells/well). Following treatments, 5-min incubation with a 0.4% w/v solution of Trypan blue in PBS was performed. Cells were released by trypsinization and counted in a Neubauer chamber.

### Apoptosis determination by TUNEL

Apoptosis was determined using the DeadEnd™ Fluorometric TUNEL System (Promega). Cells were plated in 12-well microplates (1 × 10^5^ cells/well). After the treatments, the cells were then fixed with a 4% paraformaldehyde-PBS solution for 15 min and stained with DAPI prior to detecting nuclear DNA fragmentation following manufacturer’s instructions. Photographs were taken and positive nuclei were quantified using QuPath 3.0 Software (Bankhead *et al.* 2017).

### Leydig cell tumour mouse model

An ectopic allograft model was used to evaluate curcumin *in vivo* effect on tumour growth. BALB/c × C57BL/6 (CB6F1) mice were bred at IBYME animal facility. Six-week-old male mice were subcutaneously inoculated in both flanks with 4 × 10^4^ MA-10 Leydig cells. Once the tumour reached a volume of 50 mm^3^, animals were randomly assigned to receive i.p. injections of 20 mg/kg curcumin (*n* = 6) or vehicle (*n* = 6; 10% DMSO in corn oil) every other day for 15 days. On the same days, tumour diameter was measured using a digital calliper. Tumour volume was calculated using the formula V = (*a* × *b*^2^)/2, where *a* is the longer diameter and *b* is the shorter diameter of the tumour. Body, tumour, testes, liver, and spleen weight were measured to calculate gonad, liver, and spleen somatic indexes (organ weight relative to body weight). Organs were fixed in 4% formaldehyde, paraffin-embedded, and cut into 5 μm sections for further staining with haematoxylin and eosin. Blood samples were collected and centrifuged for 15 min at 800 ***g*** for plasma separation and progesterone dosage (Architect immunoassay, Abbott). All experiments were conducted in agreement with the international guidelines and regulations of the NIH and approved by the Ethics Committee of IBYME (N° 08/2020, CICUAL – IBYME, Buenos Aires, Argentina).

### Statistical analysis

Statistical analysis was performed with the GraphPad Prism 6.0 software (GraphPad). One-way ANOVA followed by Tukey *post hoc* test was used to compare the means of multiple experimental groups. When comparing the means of two groups, a two-sided Student’s *t*-test was applied. Tumour growth curves were analysed using a two-way ANOVA, the *post hoc* Holm-Sidak test, and a non-linear regression. The *in vitro* results shown are the average of three independent experiments, and each experiment was conducted at least in triplicate.

## Results

### Curcumin stimulates MA-10 cells acute steroidogenesis and enhances StAR expression

**Figure 1:**
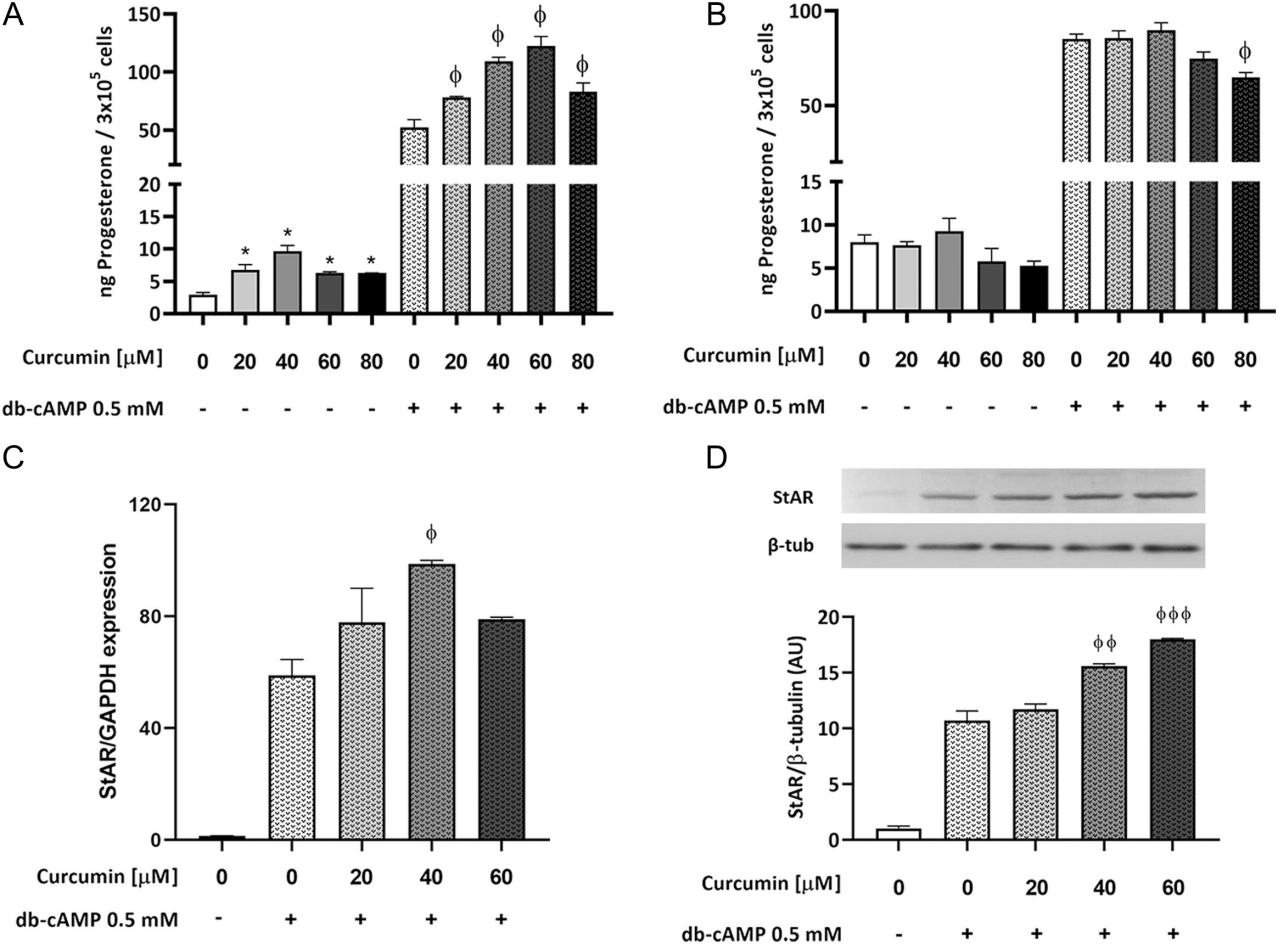
Curcumin effect on MA-10 Leydig cells steroidogenesis. The cells were incubated with increasing concentrations of curcumin (20–80 µmol/L) in the presence or absence of 0.5 mmol/L db-cAMP for 5 h (A) and 24 h (B) and progesterone was measured in the supernatants by RIA. (C) Total RNA was extracted from 5-h treated cells and RT-qPCR was performed to assess StAR mRNA levels. Relative gene expression was calculated using the relative standard curve method and data were normalized to GAPDH. (D) Protein extracts of 5-h treated cells were subjected to Western blot analysis and StAR protein levels were semi quantitated by scanning densitometry. Data were normalized to the internal control β-tubulin. Representative immunoblots are shown. Bars represent the mean ± s.e.m. of a representative (*n* = 3) triplicate experiment. *P ≤ 0.05 vs Control and ^Φ^*P* < 0.05, ^Φ^^Φ^*P* < 0.01, and ^Φ^^Φ^^Φ^*P* < 0.001 vs 0.5 mmol/L db-cAMP.

To explore the effect of curcumin on Leydig cell steroidogenesis, MA-10 cells were incubated with 20–80 µmol/L curcumin in the presence or absence of 0.5 mmol/L db-cAMP, a cell-permeable cAMP analogue that induces steroid synthesis, for 5 or 24 h. A 5-h incubation with curcumin significantly augmented progesterone production both in basal and db-cAMP-stimulated conditions (Fig. 1A). Following a 24-h incubation under the same conditions, there were no changes in unstimulated progesterone production, while there was a mild inhibition by 80 µmol/L curcumin in db-cAMP-stimulated conditions (Fig. 1B). In the presence of db-cAMP, a 5-h treatment with curcumin 40 µmol/L enhances StAR mRNA levels (Fig. 1C) and StAR protein levels (Fig. 1D), which is in line with the results observed for progesterone production.

### Curcumin affects MA-10 Leydig cells oxidative state

**Figure 2:**
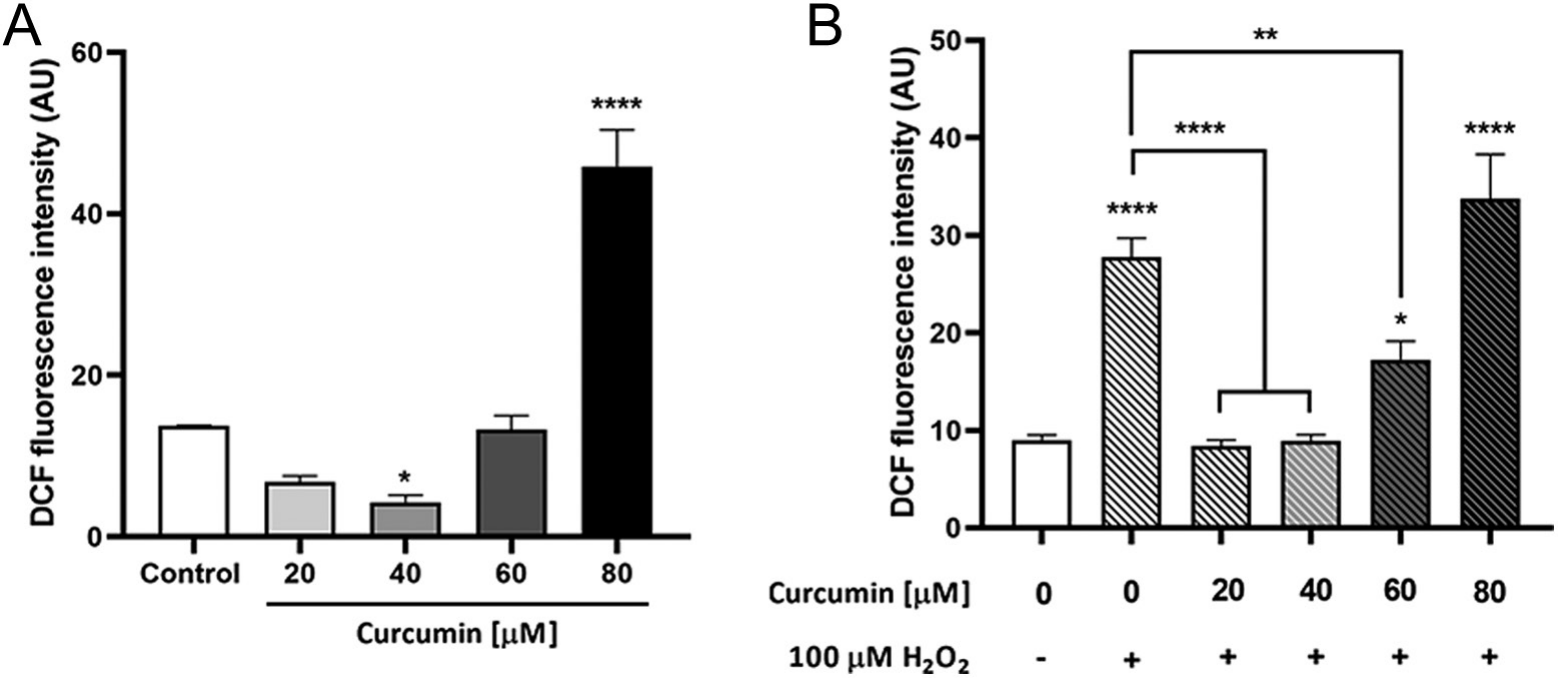
Curcumin effect on ROS production. MA-10 cells were incubated with increasing concentrations of curcumin (20–80 µmol/L) in the presence (A) or absence (B) of 100 µmol/L H_2_O_2_ for 24 h. The oxidation of DCDHF-DA to the fluorescent compound DCF was quantified as a measure of ROS production. Bars represent the mean ± s.e.m. of a representative (*n* = 3) triplicate experiment. *P ≤ 0.05, **P ≤ 0.01, ****P ≤ 0.0001 vs control or group indicated.

Curcumin antioxidant effects have been extensively reported. Besides, increased ROS levels have been associated with reduced steroidogenic capacity in Leydig cells (Hales 2002, Zhao *et al.* 2012, Wang *et al.* 2019). To assess curcumin’s effect on ROS production, MA-10 cells were incubated for 5 or 24 h with 20–80 µmol/L curcumin, and the DCF assay was performed. After a 5-h incubation, no changes in ROS production were observed in the presence of curcumin (data not shown). However, after a 24-h incubation, curcumin showed a bimodal effect on ROS production, with an inhibitory effect at low concentrations (40 µmol/L) and a stimulatory effect at the highest concentration tested (80 µmol/L) (Fig. 2A). In the presence of 100 µmol/L H_2_O_2_, 20, 40, and 60 µmol/L curcumin reverse the stimulatory effect on ROS production (Fig. 2B). This reversion is not observed with 80 µmol/L curcumin.

### Curcumin inhibits MA-10 cells proliferation and induces G2/M cell cycle arrest

**Figure 3:**
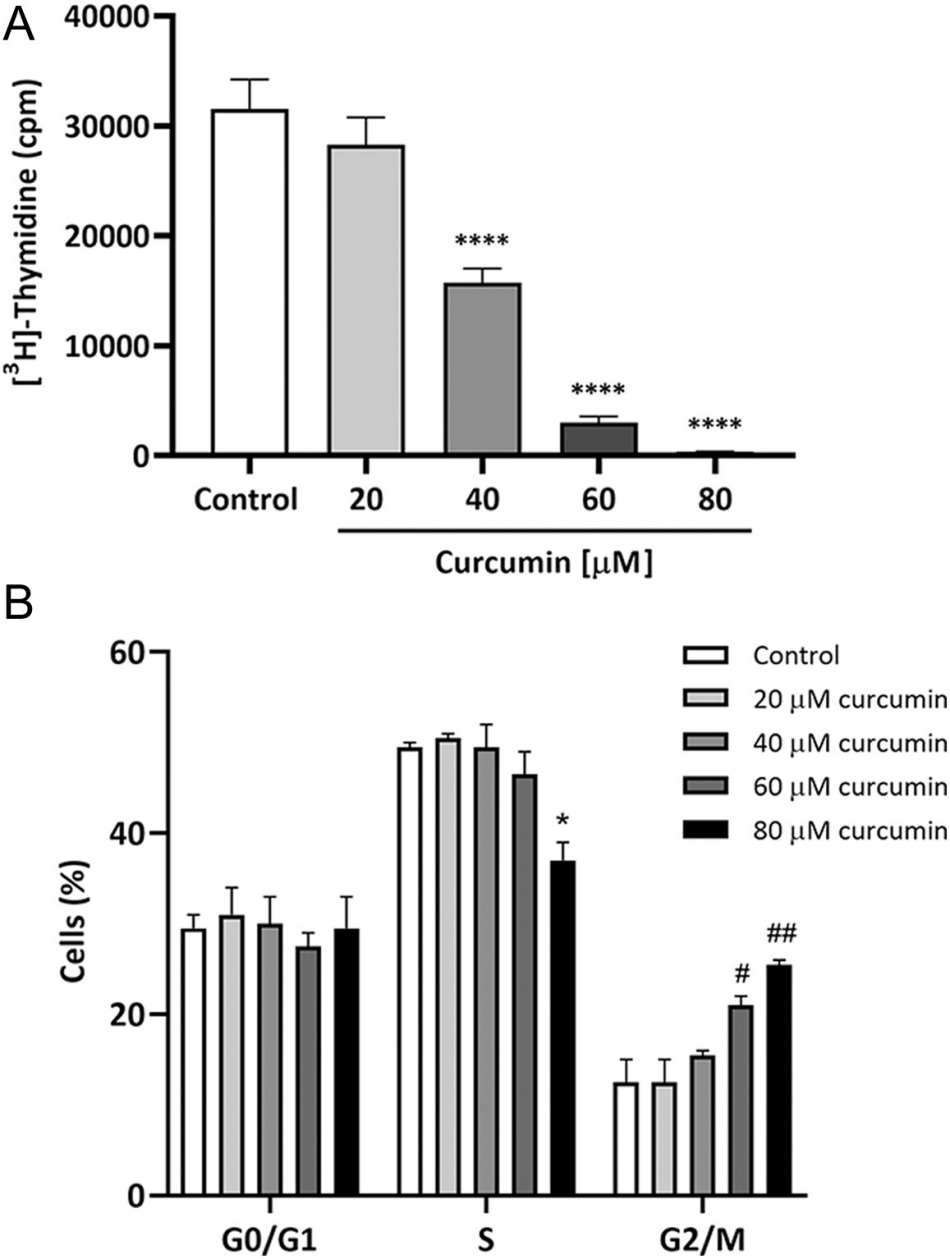
Effect of curcumin on MA-10 Leydig cells proliferation. The cells were incubated with increasing concentrations (20–80 µmol/L) of curcumin for 24 h. Cells were subjected to [^3^H]-thymidine incorporation assay (A). *P ≤ 0.05, ****P ≤ 0.0001 vs Control. To assess cell cycle distribution, cells were fixed, permeabilized, and stained with propidium iodide. DNA content was analysed by flow cytometry. The percentage of cells in the G1/G0, S, and G2/M phases was obtained (B). *P ≤ 0.05 vs control (S phase). ^#^*P* ≤ 0.05, ^##^*P* ≤ 0.01 vs Control (G2/M phase). Bars represent the mean ± s.e.m. of a representative (*n* = 3) octuplicate experiment.

Curcumin cytostatic properties were evaluated on MA-10 Leydig cells. For this purpose, they were incubated with increasing concentrations of curcumin (20–80 µmol/L) for 24 h and [^3^H]-thymidine incorporation assay was performed. Curcumin inhibition of MA-10 cell proliferation in a concentration-dependent manner is depicted in Fig. 3A, with a maximum effect observed at 80 µmol/L curcumin (*P* ≤ 0.0001). Having observed curcumin’s negative effect on MA-10 Leydig cells’ proliferative capacity, we sought to determine its effect on cell cycle progression. Therefore, MA-10 cells treated under the same conditions as those used for the proliferation assays were subjected to propidium iodide staining and DNA quantitation by flow cytometry. Figure 3B shows a significant increase in the percentage of cells in the G2/M phase induced by 60 and 80 µmol/L curcumin and a reduction in the percentage of cells in S phase by 80 µmol/L curcumin. To unveil the molecular mechanisms underlying this arrest, MA-10 cells were incubated for 12 and 24 h with 40 and 60 µmol/L curcumin and p21^Cip1^, cyclin D1 (CCND1) and p27^Kip1^ mRNA levels were assessed. p21^Cip1^ expression is significantly enhanced and cyclin D1 expression is significantly reduced by 60 µmol/L after 12-h incubation (data not shown). This effect on these cell cycle regulators mRNA levels is not observed after a 24-h treatment.

### Curcumin reduces MA-10 cells viability and induces apoptosis

**Figure 4:**
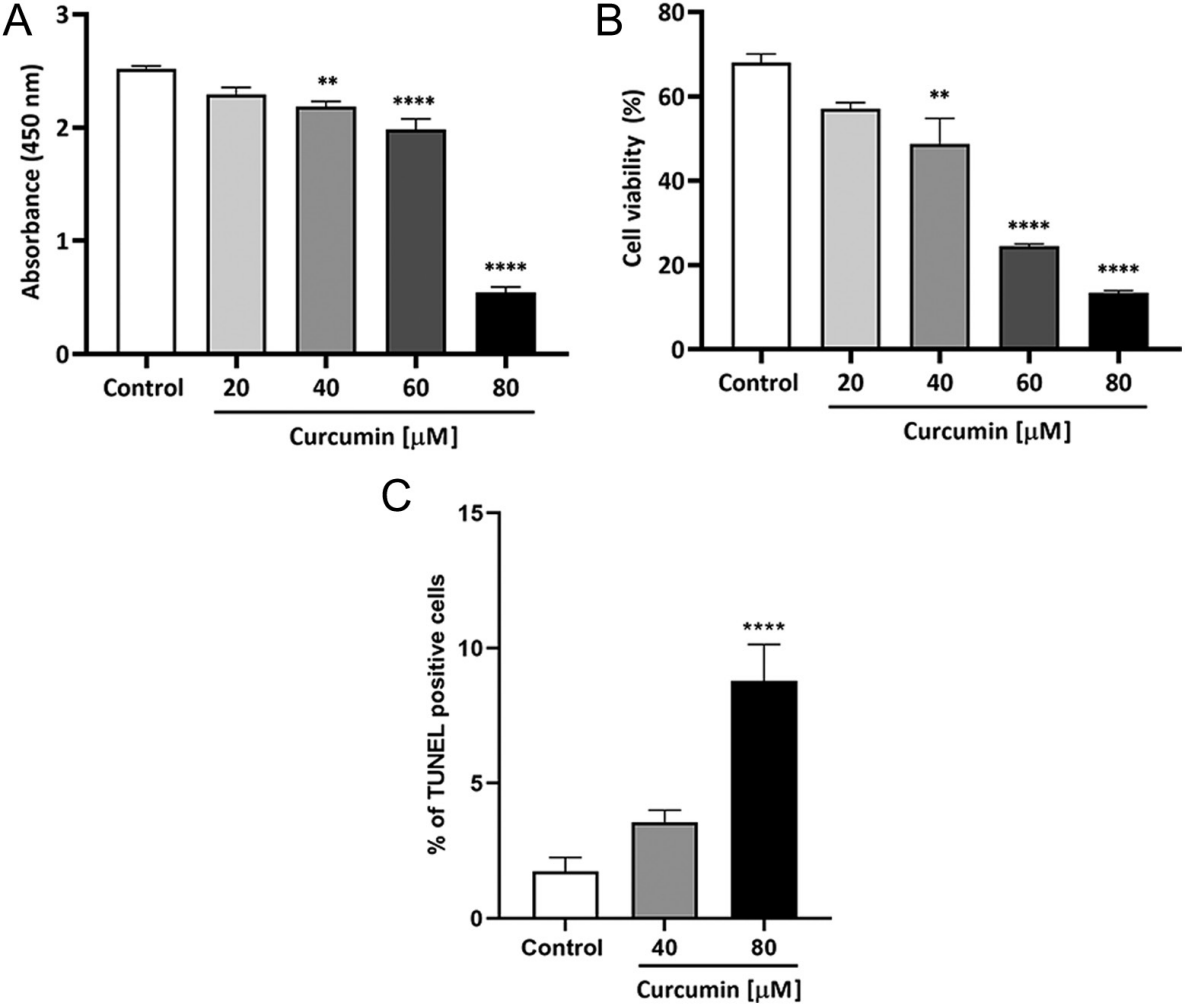
Effect of curcumin on Leydig cells viability and apoptosis induction. MA-10 cells were incubated with increasing concentrations (20–80 µmol/L) of curcumin for 24 h. Then, they were subjected to the WST (A) and Trypan blue (B) assays. To assess the activation of the apoptotic pathway, cells were incubated for 24 h with 40 and 80 µmol/L curcumin. Then, they were fixed and the TUNEL assay was performed. The percentage of TUNEL-positive cells is shown (C). Bars represent the mean ± s.e.m. of a representative (*n* = 3) triplicate experiment. **P ≤ 0.01, ****P ≤ 0.0001 vs control.

To evaluate the effect of curcumin on cell viability, MA-10 Leydig cells were incubated with increasing concentrations of curcumin (20–80 µmol/L) for 24 h and the WST-1 and the Trypan blue exclusion assays were performed. Trypan blue exclusion was first assessed after 5-h incubation, but no differences were observed among treatments (data not shown). After a 24-h exposure to 40–80 µmol/L curcumin, MA-10 cell viability was reduced in a concentration-dependent manner (Fig. 4A and B). To determine whether this reduction in cell viability could be due to the activation of the apoptotic programme of cellular death, MA-10 cells were treated with 40 and 80 µmol/L curcumin for 24 h and the TUNEL assay was performed. As shown in Fig. 4C, 80 µmol/L curcumin significantly augments the percentage of apoptotic cells.

### Curcumin reduces Leydig cell tumour growth

**Figure 5:**
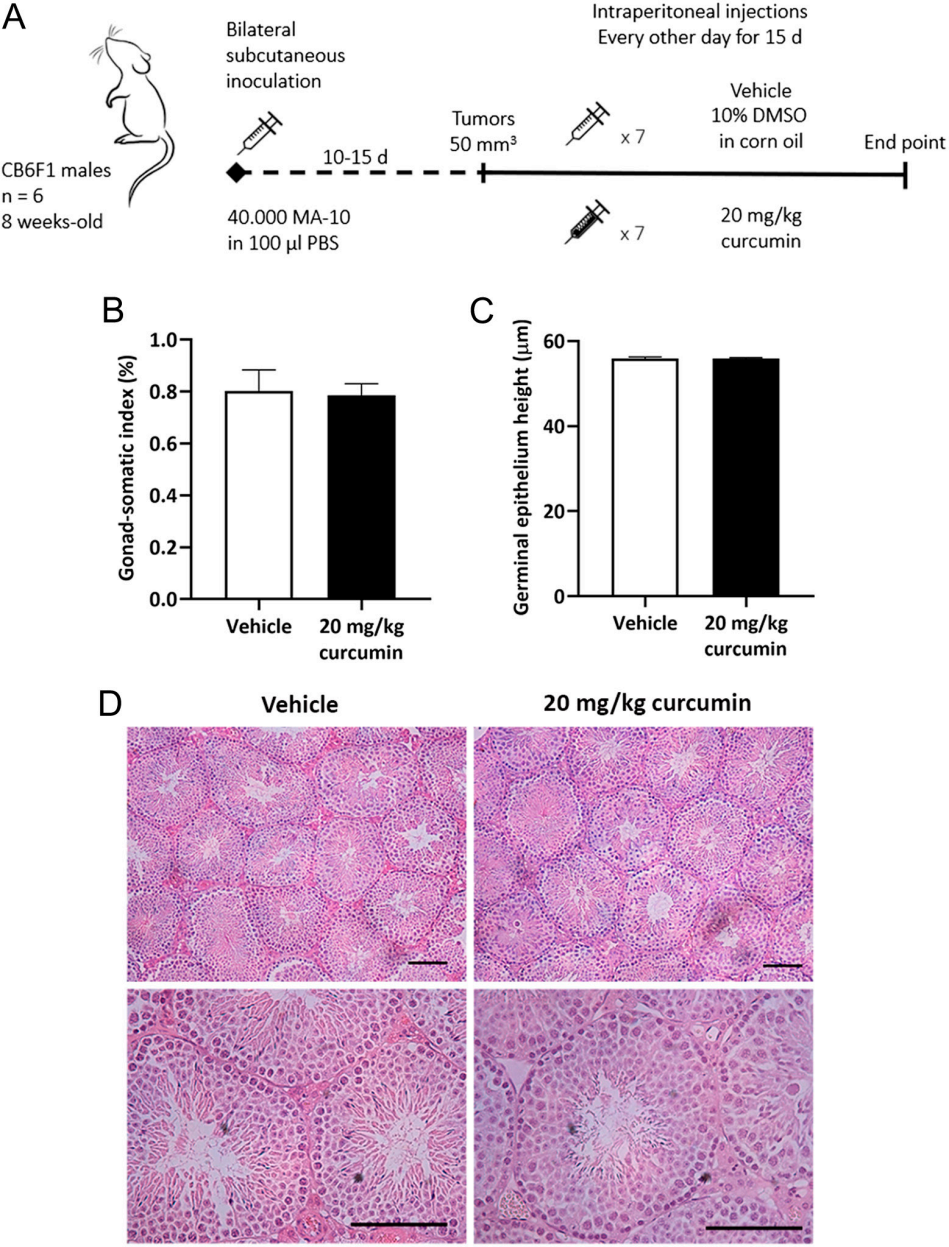
*In vivo* assessment of curcumin effects on testicular integrity. (A) Experimental treatment scheme of the ectopic allograft Leydig cell tumour murine model. At the endpoint, testes were weighed to calculate the gonad-somatic index (B). Testes were fixed in 4% formaldehyde, paraffin-embedded, and stained with haematoxylin and eosin. Light micrography photographs were obtained and 200 round or nearly round cross-sections of the seminiferous tubules from each mouse were randomly analysed to measure germinal epithelium height (C). Bars represent the mean ± s.e.m.. Representative photographs are shown in (D). Scale bars represent 50 µm.

**Figure 6:**
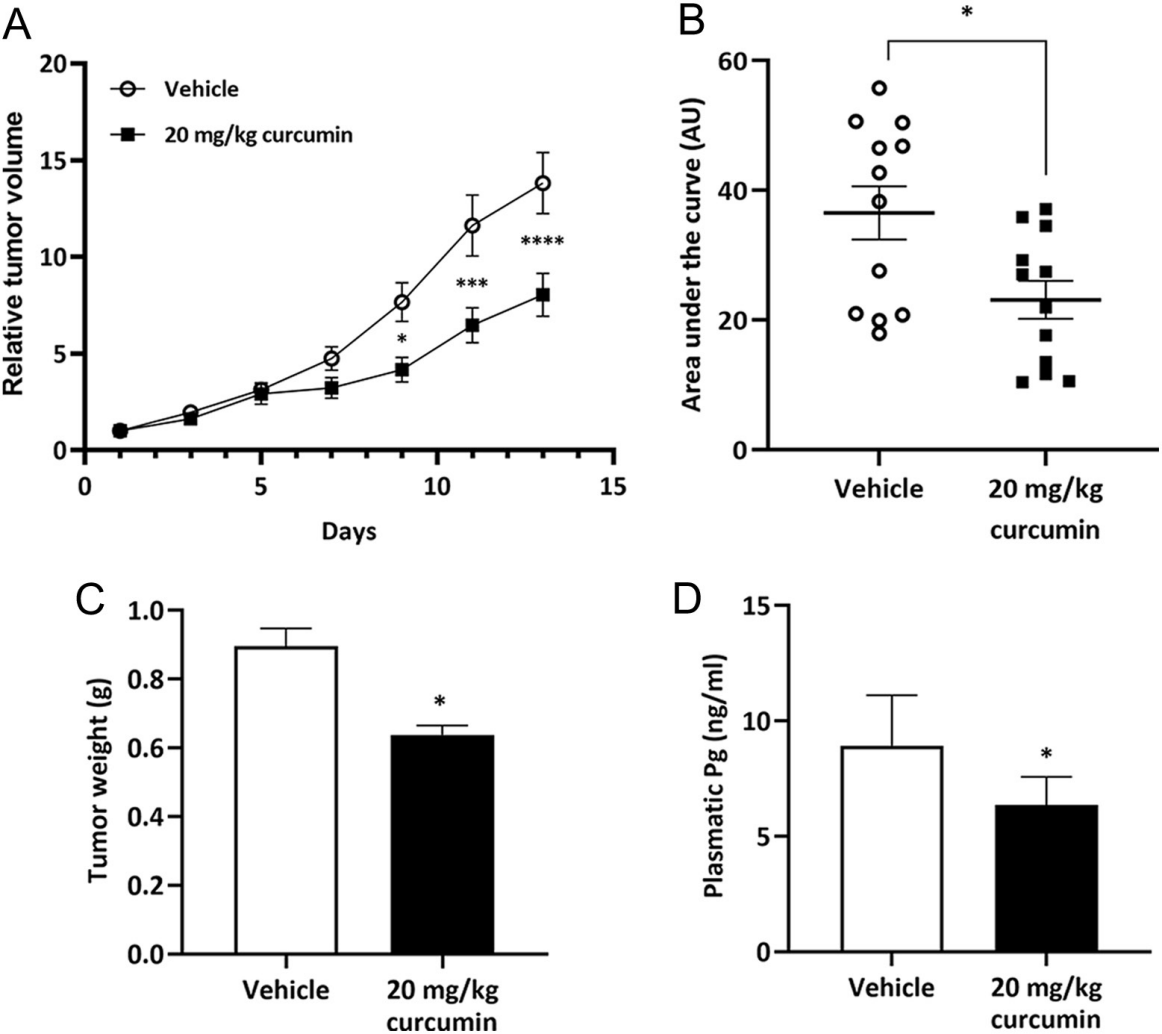
Effect of curcumin treatment on MA-10 Leydig cell tumour growth. MA-10 cells were injected in both flanks of CB6F1 mice. Once tumours reached a volume of 50 mm^3^, animals were randomly assigned to receive i.p. injections of 20 mg/kg curcumin (*n* = 6) or vehicle (*n* = 6; 10% DMSO in corn oil) every other day for 15 days. On the same days, tumour diameter was measured using a digital calliper, and tumour volume was calculated (A). The area under the curve was also calculated for each group (B). Tumour weight (C) and plasmatic progesterone (Pg) concentration (ng/mL) (D) were determined at the endpoint. Each point or bar represents the mean ± s.e.m.. *P < 0.05, ***P > 0.001, ***P < 0.001 vs vehicle.

Having unveiled curcumin’s significant *in vitro* effects on MA-10 Leydig cells steroidogenesis, redox status, proliferation, and viability, we then sought to assess whether these actions could translate into an *in vivo* effect on Leydig cell tumour growth. For this purpose, a syngeneic mouse model was employed. CB6F1 mice were subcutaneously inoculated with MA-10 Leydig cells to generate ectopic Leydig cell tumours. Once tumours were established, animals were randomly assigned to receive i.p. injections of 20 mg/kg curcumin or vehicle every other day for 15 days (treatment scheme in Fig. 5A). On the same days, animals were treated, tumour diameter was measured, and tumour volume was calculated. First, the general state of health parameters was evaluated. Curcumin treatment did not affect the animals’ body weight. Also, there were no differences in spleen and liver somatic indexes between vehicle and curcumin-treated animals. Liver histology was not altered by curcumin (data not shown). As mentioned earlier, conventional cancer treatments may have severe effects on testicular architecture and function. For this reason, we evaluated whether curcumin treatment affected testicular integrity. In this respect, we observed no differences between treatments in the gonad-somatic index, the height of the germinal epithelium, and the histology of the testis (Figs. 5B, C and D). As regards tumour growth, curcumin significantly reduced tumour volume from treatment day 9 on (Fig. 6A) as evidenced by a difference in the areas under the growth curve between groups (Fig. 6B). Besides, curcumin-treated animals exhibited a higher tumour doubling time (5.332 days, CI = 4.296–7.028) than vehicle-treated animals (4.75 days, CI = 3.899–6.076). In line with these results, tumour weight and plasmatic progesterone levels at the end point were significantly lower in curcumin-treated group (Fig. 6C and D). There were no differences between groups in plasmatic progesterone levels when the absolute values were relativized to tumour weight.

## Discussion

Humankind has been consuming curcumin for centuries, mostly driven by its multiple beneficial health effects. According to a WHO/FAO report (1974), the daily dietary intake of turmeric within populations that traditionally use it as a culinary spice ranges between 2 and 2.5 g, which corresponds to 60–100 mg of curcumin (Shah *et al.* 1999). To further exploit its benefits, enriched extracts and over-the-counter supplements with a 10-fold higher concentration of curcumin are being used. Most curcumin-containing products tested in phase I and II clinical trials reach doses ranging from 1000 to 8000 mg of curcumin per day, being 8000 mg/day the maximum tolerable dose that does not cause serious adverse events (National Cancer Institute 2022). The growing interest in its beneficial health effects has led to increasingly widespread use of this natural product. For these reasons, and with a view to furthering the knowledge regarding curcumin’s actions on different cell types, we sought to assess the effects of this natural compound on the endocrine cell population of the testis, which have been scarcely researched.

For the first set of *in vitro* experiments, we exposed MA-10 Leydig cells to curcumin and observed significant effects from the 20 µM concentration. We then confirmed that this concentration can be reached* in vivo* in animals and healthy volunteers (Vareed *et al.* 2008), especially when curcumin is co-administered with other drugs or incorporated in carriers such as microspheres that augment its bioavailability (Shoba *et al.* 1998, Karade & Jadhav 2018). In the present report, we first show that curcumin concentration dependently stimulates MA-10 Leydig cells’ acute steroidogenesis *in vitro*, both in the presence and absence of db-cAMP. The increase in progesterone levels is accompanied and can be partly explained, by an increase in StAR protein and mRNA levels. After a 5-h treatment with 60 µM curcumin, we observed an increase in protein, but not in StAR mRNA levels. This could be explained considering that once StAR protein and progesterone levels reach a certain level and a feedback response is triggered, mRNA levels are downregulated. To date, there is only one other article reporting the *in vitro* effects of curcumin on MA-10 steroid release. Lin and colleagues’ data show that 10–80 µmol/L curcumin suppress 8-Br-cAMP-induced steroidogenesis in MA-10 and primary mouse Leydig cells by inhibiting the expression of StAR and Cyp11a1 (Lin *et al.* 2018). Nonetheless, the source of the drug is not cited in the article, so it can be speculated that the differing results might be due to differences in quality, purity, or even chemical nature of the drug. Chen and colleagues have shown that 20 µmol/L curcumin do not affect basal testosterone levels of TM3 mice Leydig cells, but it revert testosterone production inhibition by palmitic acid (Chen *et al.* 2019). This protective effect against endocrine disruption by a variety of stressors has also been observed *in vivo*. It has been reported that a decrease in serum testosterone levels induced by scrotal hyperthermia (Afshar *et al.* 2021), heavy metals such as cadmium (Yang *et al.* 2019), and gallic acid (Abarikwu *et al.* 2014), among others, can be reversed when the animals are co-treated with curcumin. In these two last reports, animals even show an increase in circulating testosterone levels when treated with curcumin alone. It has also been reported that curcumin stimulates ovarian granulosa cells’ steroidogenesis *in vitro* (Kádasi *et al.* 2017).

There is plenty of evidence supporting curcumin’s capacity to preserve testicular architecture and function, which is usually attributed to its antiapoptotic and antioxidant actions. *In vitro* analyses on Leydig cell lines have indicated that curcumin exerts a protective effect against a variety of stressors mainly by suppressing apoptosis and also alleviating oxidative stress and endoplasmic reticulum stress (Chen *et al.* 2019, 2020, Yildizbayrak & Erkan 2019). When assessed *in vivo*, curcumin’s protective role translates into preserved testicular volume, tubular epithelium height, tubule length, and number of viable Leydig and germ cells (Noorafshan *et al.* 2011, Lin *et al.* 2015, Mahmoudi *et al.* 2017, Chen *et al.* 2019, Khosravi *et al.* 2020, Afshar *et al.* 2021). In the present report, we show that curcumin reduces basal and H_2_O_2_-stimulated ROS levels in MA-10 Leydig cells at low concentrations. In line with these results, curcumin has been proposed as a modulator of the Nrf2 pathway in the testis, a transcription factor that regulates the expression of several antioxidant enzymes (Ashrafizadeh *et al.* 2020). An *in vivo* study in rats has shown that, in the presence of the stressor di-(2-ethylhexyl) phthalate, curcumin increases Nrf2 levels in the testis and ameliorates testicular injury in rats (Abd El-Fattah *et al.* 2016). Additionally, due to the reactivity of its diketone moiety and its phenolic groups, which undergo oxidation by electron transfer and hydrogen abstraction, curcumin has shown direct ROS scavenging effects (Priyadarsini 2014). In contrast with this protective role, our results indicate that 80 µmol/L curcumin augment basal ROS levels and do not reverse H_2_O_2_ stimulatory effect. We suggest that this effect reflects the incipient activation of cell death programmes, which is accompanied by cellular stress and redox imbalance.

Extensive evidence has supported the potential role of curcumin in modulating carcinogenesis. Among other pathways, it has proven to interfere with proliferation, cell cycle, apoptosis, and cell survival (National Cancer Institute 2022). In the present report, we show that curcumin reduces MA-10 Leydig cells’ proliferative capacity and induces G2/M cell cycle arrest. We have also shown that curcumin reduces MA-10 Leydig cells’ viability and that it significantly augments the percentage of apoptotic cells at the highest concentrations. As previously discussed, curcumin has also proven to have a protective antiapoptotic capacity in other experimental models, so it can be speculated that the effect on this pathway depends both on the concentration of the drug and the cell type under study. In a report by Chen and colleagues, TM3 cells, a Leydig cell line derived from immature 11- to 13-day mouse testes, were treated with curcumin for 24 h, and the concentration of 40 µmol/L negatively affected cell viability (Chen *et al.* 2019). The induction of G2/M and cell cycle arrest by curcumin was also observed in other cancer cell types, such as human osteosarcoma (Lee *et al.* 2009), pancreatic (Zhu & Bu 2017), acute myeloid leukaemia (Zhou *et al.* 2021), and colon adenocarcinoma cells (Agarwal *et al.* 2018), among others. It has also been reported that a curcumin derivative, curcumin nicotinate, inhibits proliferation of colon, breast, and nasopharyngeal cancer cells, induces apoptosis and cell cycle arrest at G2/M phase through a p53-mediated mechanism, and p21^Cip1^ and pro-apoptotic proteins upregulation (He *et al.* 2019). In the present report, we observed this induction of p21^Cip1^ expression in MA-10 Leydig cells, which can explain the arrest in G2/M due to the participation of this protein in the thoroughly described nuclear accumulation of inactive cyclin B1-Cdk complexes (Ando *et al.* 2001, Charrier-Savournin *et al.* 2004, Müllers *et al.* 2014). We also report a reduction in cyclin D1 mRNA levels, which could impair cell cycle progression by hindering the exit from G1, which in turn reduces the percentage of cells in S. It should be noted that, presumably due to its multiplicity of molecular targets, it has also been reported that curcumin induces cell cycle arrest in G0/G1 (Rajitha *et al.* 2016, Sha *et al.* 2016) and G1/S (Shishodia *et al.* 2005) in other cell types.

Finally, and observing the strong *in vitro* antiproliferative capacity of curcumin on MA-10 Leydig cells, we examined its effect on a syngeneic Leydig cell tumour mouse model. The *in vivo* curcumin dose was determined following the estimation proposed by Lindl and colleagues (Lindl *et al.* 2005) based on the observed *in vitro* effective concentration, and comparing this estimation with the doses used in preclinical and clinical studies (Basu *et al.* 2021). In the *in vivo* model, we first discarded the detrimental effects of curcumin on general health state parameters such as body weight, liver and spleen-somatic indexes, and liver histology. Besides, we observed that curcumin did not affect testicular integrity, which is particularly relevant considering the importance that is currently being given to the preservation of reproductive function, especially in paediatric cancer patients (Johnson *et al.* 2017). Lastly, we described that 20 mg/kg of curcumin significantly reduces Leydig cell tumours volume and weight and progesterone plasmatic levels. Noticeably, when progesterone plasmatic levels were relativized to tumour weight, no differences were observed between groups. Though it is usually expectable to obtain different results in whole animal experiments than those of the *in vitro* assays, further research should be done to describe curcumin’s effects on mice steroidogenic function. In this context, it cannot be ruled out that the absence of progesterone induction *in vivo* could be explained by negative feedback due to an increase in testosterone levels in response to curcumin treatment. Besides, according to the *in vitro* results, curcumin modulates acute steroidogenesis, so it is possible that this effect is not detectable after a 15-day *in vivo* treatment.

Though further experiments should be performed, both in normal Leydig cells and in other Leydig cell lines to better represent the wide spectrum of Leydig cell tumours, our results constitute the first evidence of curcumin’s antitumour effect on Leydig cell tumours and supports the vast number of reports showing its favourable effects both in preclinical and clinical trials that propose it as an anticancer adjuvant. Curcumin has evidenced anticancer potential in clinical trials on breast, colorectal, prostate, cervical cancer and leukaemia, and in preclinical trials on several other types of cancer (Patel *et al.* 2020). In view of its clinical potential, there are two main aspects to take into consideration when researching curcumin’s *in vivo* effects. First, there is a striking lack of clarity in relation with the chemical nature of curcumin-containing products used in the studies, which ranges from raw *C. longa* rhizomes (turmeric) to curcuminoid-enriched extracts to pure curcumin. On the other hand, curcumin is a highly hydrophobic molecule, chemically unstable, and with a poor pharmacokinetic profile. For this reason, there is plenty of research aiming at the development of analogues that maintain curcumin’s beneficial health effects while improving its pharmacokinetic profile and bioavailability (Dei Cas & Ghidoni 2019). Interestingly, there are two opposing views regarding the use of curcumin as a therapeutic alternative. Detractors place curcumin in the group of pan assay interference compounds and invalid metabolic panaceas. These compounds are criticized because the descriptions of their multiple effects are based solely on the foundational premise of the reported activity and their therapeutic utility without further precision on the medicinal chemistry of their actions (Nelson *et al.* 2017). But there are others who state that cancer being such a complex disease, caused by the dysregulation of a variety of genes, agents that target multiple gene products are needed for its prevention and treatment (Kunnumakkara *et al.* 2008). Regardless of this discussion and considering the growing popularity and widespread consumption of curcumin, there is no doubt that its effects should be thoroughly assessed.

To sum up, curcumin has proven to have direct effects on MA-10 Leydig cells’ functions. It positively regulates steroid release, at least partly through the upregulation of StAR protein, while modulating their oxidative state. *In vitro* assays also show that curcumin at high concentrations inhibits cell proliferation, inducing G2/M cell cycle arrest, and reduces cell viability, activating the apoptotic cell death pathway. Finally, this report adds novel evidence on curcumin’s anticancer effect on Leydig cell tumours, which emerges as a natural therapeutic option to be used as an adjuvant of conventional treatments that does not have a negative impact on testicular integrity.

## Declaration of interest

The authors declare that there is no conflict of interest that could be perceived as prejudicing the impartiality of the research reported.

## Funding

This investigation was supported by grants from the Agencia Nacional de Promoción Científica y Tecnológica (ANPCyT, PICT 2015 No. 2650 and PICT 2018 No. 1973).
